# Development of a UK core dataset for geriatric medicine research: a position statement and results from a Delphi consensus process

**DOI:** 10.1186/s12877-023-03805-5

**Published:** 2023-03-23

**Authors:** Carly Welch, Daisy Wilson, Avan A. Sayer, Miles D. Witham, Thomas A. Jackson, on behalf of the UK Geriatric Medicine Core Dataset Extended Working Group, Raj Rajkumar, Jugdeep Dhesi, Mary Ni Lochlainn, Terry Aspray, Richard Dodds, James Frith, Sarah Richardson, Ellen Tullo, Alison Yarnall, Richard Walker, Emma Cunningham, Josephine Prynn, Harnish Patel, Divya Tiwari, Stephen Makin, Phyo Myint, Emily Henderson, Victoria Keevil, Katherine Walesby, Louise Allan, Jane Masoli, Terry Quinn, Andrew P. Clegg, Matthew Hale, Simon Conroy, Joanne Taylor, John Gladman, Adam Gordon, Rowan Harwood, Natalie Cox, Helen Roberts

**Affiliations:** 1 Medical Research Council – Versus Arthritis Centre for Musculoskeletal Ageing Research, University of Birmingham and University of Nottingham, B15 2TT, Birmingham, UK; 2 Institute of Inflammation and Ageing, College of Medical and Dental Sciences, University of Birmingham, B15 2TT, Birmingham, UK; 3 University Hospitals Birmingham NHS Foundation Trust, B15 2GW, Birmingham, UK; 4 Guy’s and St Thomas’ NHS Foundation Trust, St Thomas’ Hospital, Westminster Bridge, London, SE1 7EH UK; 5 AGE Research Group, NIHR Newcastle Biomedical Research Centre, Newcastle University and Newcastle upon Tyne Hospitals NHS Foundation Trust, Newcastle, UK

**Keywords:** Minimum dataset, Frailty, ADL, CFS, Barthel

## Abstract

**Background:**

There is lack of standardisation in assessment tools used in geriatric medicine research, which makes pooling of data and cross-study comparisons difficult.

**Methods:**

We conducted a modified Delphi process to establish measures to be included within core and extended datasets for geriatric medicine research in the United Kingdom (UK). This included three complete questionnaire rounds, and one consensus meeting. Participants were selected from attendance at the NIHR Newcastle Biomedical Research Centre meeting, May 2019, and academic geriatric medicine e-mailing lists. Literature review was used to develop the initial questionnaire, with all responses then included in the second questionnaire. The third questionnaire used refined options from the second questionnaire with response ranking.

**Results:**

Ninety-eight responses were obtained across all questionnaire rounds (Initial: 19, Second: 21, Third: 58) from experienced and early career researchers in geriatric medicine. The initial questionnaire included 18 questions with short text responses, including one question for responders to suggest additional items. Twenty-six questions were included in the second questionnaire, with 108 within category options. The third questionnaire included three ranking, seven final agreement, and four binary option questions. Results were discussed at the consensus meeting. In our position statement, the final consensus dataset includes six core domains: demographics (age, gender, ethnicity, socioeconomic status), specified morbidities, functional ability (Barthel and/or Nottingham Extended Activities of Daily Living), Clinical Frailty Scale (CFS), cognition, and patient-reported outcome measures (dependent on research question). We also propose how additional variables should be measured within an extended dataset.

**Conclusions:**

Our core and extended datasets represent current consensus opinion of academic geriatric medicine clinicians across the UK. We consider the development and further use of these datasets will strengthen collaboration between researchers and academic institutions.

**Supplementary Information:**

The online version contains supplementary material available at 10.1186/s12877-023-03805-5.

## Introduction

Research in geriatric medicine is an expanding field [1, 2]. However, within the United Kingdom (UK), research is predominantly centred within several large university institutions. There is a limited culture of research within clinical geriatric medicine outside of these institutions [1, 2]. Similar patterns have been described internationally [1]. Where research is conducted, this is largely small and single centre, with minimal opportunity for collaboration between sites, although initiatives such as the Geriatric Medicine Research Collaborative have assisted to overcome this [3]. Investigators will often devote a large amount of time reviewing and deciding upon which assessment tools to use. Without a standardised approach, discrepancies in assessment of an already heterogeneous population mean that datasets cannot be easily compared or connected.

The Gerontonet collaboration previously aimed to develop a geriatric minimum dataset for clinical trials in Europe [4]. However, this has been rarely utilised in clinical research within the UK due to limited dissemination, and concerns about applicability to all geriatric medicine research and burden of multiple assessments with the 25 items included. Additionally, there were concerns that this should be updated with use of items such as the Charlson index considered outdated [5], the Mini-Mental State Examination (MMSE) being copyright protected [6], and newer tools such as the Clinical Frailty Scale (CFS) [7] not included. Separately, the Canadian Frailty Network is undertaking an initiative to decide upon core data elements and core outcome measures for frailty research [8]. The Core Outcome Measures in Effectiveness Trials (COMET) initiative has been developed as a means to reach consensus on outcome measurements that should be reported for clinical trials and demonstrates the benefits of standardisation across research [9]. Whilst the COMET initiative has traditionally focused on outcomes, we consider that standardisation of data collection tools in themselves to be of value, to improve quality of research, improve efficiency in protocol development, and enable collaboration and comparison of datasets from research from different sites. The Geriatric Expert Group of the European Medicines Agency has produced guidance on instruments for baseline characterisation of physical frailty in older populations in clinical trials [10], but such guidance may not be applicable to all study designs and research questions, such as observational studies in hospitalised older adults or those who are nursed in bed.

## Aims

1. To develop a core dataset for use in prospectively conducted geriatric medicine research.
2. Provide a position statement to guide researchers as to how the core dataset domains should be measured.

## Methods

**Fig. 1:**
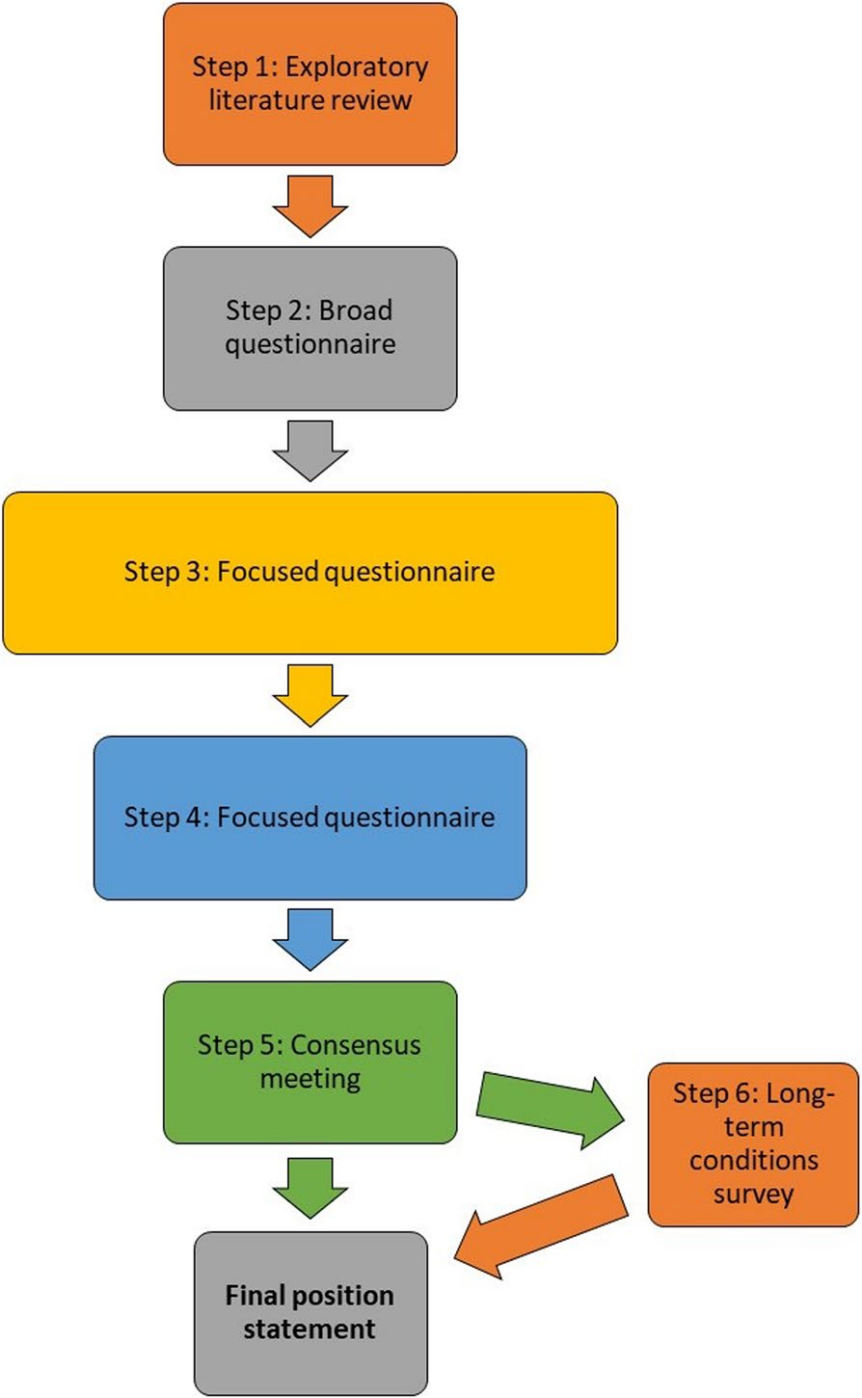
Steps involved in Delphi process in developing final position statement

We used a modified Delphi approach to obtain a consensus opinion on the items to be included within the core dataset (Fig. 1). At each round, this consisted of UK academic geriatricians, including experienced consultant principal investigators, early career researchers/ trainees, and nurses/ allied health professionals/ pharmacists. The expertise of participants was broad within the field of geriatric medicine research, in terms of both research fields, and methodologies (Additional file 1).

### Delphi step 1 – literature review and discussion

An initial exploratory literature review of the literature was conducted to identify common themes included within prospective clinical studies within geriatric medicine research, and measures available (DW, CW, TAJ). Prospective cross-sectional, cohort, and controlled studies within geriatric medicine research conducted in the UK in the previous 5 years (1st Jan 2014 – 31st Dec 2018) were identified from publications in high impact geriatric medicine journals. The approach to identification and exclusion of articles was non-systematic. The results of this and the proposed minimum dataset were presented at the National Institute for Health Research (NIHR) Newcastle Biomedical Research Centre (BRC) academic geriatric medicine event in May 2019 [1].

### Delphi step 2 – broad questionnaire

The results of the literature review, and discussion at the NIHR BRC Newcastle event were used to generate a questionnaire with broad responses (Questionnaire 1, Additional file 2). This questionnaire was distributed to all delegates who had attended the NIHR BRC meeting, and additional expert academic geriatricians. Participants were asked (via short answer questions with no prespecified responses) to comment on the proposed items to be included within the minimum dataset, and how these should be measured. They were also given the opportunity to suggest additional items that should be included, that had not been included in the survey.

In evaluating the responses given at this step, it became clear there were two separate elements to the proposed dataset:

1. Identifying measures that should be considered “core” to all prospectively conducted geriatric medicine research, and how these should be measured.
2. Standardising measurement of additional/ optional elements to research, to enable sharing of datasets across studies and comparisons where these are measured.

Subsequent steps of the Delphi process were, therefore, designed considering both of these elements, as described below.

### Delphi step 3 – focused questionnaire

All measures that were suggested in Step 2 were included as options within the Step 3 focused questionnaire (Additional file 2). This survey was distributed to everyone who had been invited to participate in Step 2. Participants were asked to declare at this stage whether they considered each aspect of assessment to be “core” to geriatric medicine research, regardless of how it was to be measured. Participants were asked to select single responses for the most appropriate assessment tool and method from each of the options included for each aspect of assessment, regardless of whether or not they selected this item as “core”.

### Delphi step 4 – focused questionnaire

The responses from the Step 3 questionnaire were utilised to generate a further focused questionnaire in Step 4 (Additional file 2). If more than 80% of participants in Step 3 had considered an item core, then this item was proposed as a core item in Step 4, and participants were invited to express their agreement with this. Items that were considered not relevant to the minimum dataset (either core or extended option items) by more than 75% of participants in Step 3 were removed in Step 4. If a measure was proposed by more than 80% in Step 3, participants were invited to express their agreement with this in Step 4. If there was a divide in opinion between two measures, participants were asked to select one of these in Step 3. If there was a divide in opinion between more than two measures, participants were asked to rank these options. The mean ranking was calculated across participants. This questionnaire was distributed more widely, and responses were completely anonymous. As well as those invited to previous rounds, this survey was also distributed to members of the UK Association of Academic Geriatric Medicine, the British Geriatrics Society Research and Academic Development Committee, and via a national mailing list of research active clinicians in Geriatric Medicine, and early career researchers.

### Delphi step 5 – consensus meeting

A consensus meeting was held virtually on November 5th 2020. Participants were invited via the same channels used to distribute the survey from Step 4. The results of earlier rounds were presented to all attendees, and each item was discussed until a consensus decision was made. All participants were given the opportunity to contribute. This included both senior academic geriatricians and early career researchers/ trainees.

### Delphi step 6 – multiple long-term conditions survey

During the consensus meeting, it was felt that the survey responses from the earlier rounds were insufficient to make a consensus decision about the recording of long-term conditions within the core dataset. An agreement was made for a wider distribution of a final survey, asking clinicians to rank their perceived importance of 34 pre-specified conditions from 1 = most important, to 34 = least important. The ten conditions with the lowest mean ranking score were then chosen to be included within the core dataset for geriatric medicine research (Additional file 1).

## Results

The full list of items included within each step of the modified Delphi process is available online (Additional file 3). Twenty-nine delegates attended the initial NIHR BRC meeting in Newcastle, May 2019. The initial literature review identified thirteen possible domains to be included within the core dataset, which were included within the first questionnaire. Nineteen responses were obtained from this. Five additional items were suggested at this stage and were included within the second questionnaire without prespecified suggestions as to how these should be measured. A total of 26 items were proposed within the second questionnaire (Step 3). All options suggested from the first questionnaire (Step 2) were included with a total of 108 individual items. Twenty-one responses were obtained from this questionnaire. Of the 26 proposed items, 7 were considered to be core by over 80% of respondents. Seven items were considered to be core by less than 25% of respondents, and a decision was made to remove these items at this stage. Fifty-eight responses were obtained from the third questionnaire (Step 4). Thirteen collaborators attended the consensus meeting (Step 5), and eighteen responses were obtained from the morbidities survey (Step 6).

Aspects of the dataset that are considered required for all geriatric medicine research have been included within the core dataset (Table 1). Assessments that are considered important but not required or practical for all research have been included within the extended dataset (Table 2). In the extended dataset, we make recommendations as to how these should be recorded to ensure standardisation where these are measured. It should be noted that aspects of the extended dataset are strongly encouraged where possible, but dependent on the design and setting of the study research may still continue and be of value without these. To improve both consistency of data collection, and reduce burden of study set-up/design, we have produced template Case Report Forms (CRFs) that can be adapted as per the requirements of the respective research (Additional files 4 and 5).

**Table 1:** Core dataset criteria and guidance for scoring/ completion

	Criteria	Guidance
Demographics	• Age • Gender • Ethnicity • Place of residence • Socioeconomic status	Ethnicity: UK census categories: https://www.ethnicity-facts-figures.service.gov.uk/style-guide/ethnic-groups Socioeconomic status: England – English indices of deprivation: https://imd-by-postcode.opendatacommunities.org/imd/2019 Scotland – Scottish index of multiple deprivation: https://www.gov.scot/publications/scottish-index-of-multiple-deprivation-2020v2-postcode-look-up/ Wales – Welsh index of multiple deprivation: https://statswales.gov.wales/Catalogue/Community-Safety-and-Social-Inclusion/Welsh-Index-of-Multiple-Deprivation Northern Ireland – Northern Ireland multiple deprivation measure https://deprivation.nisra.gov.uk/
Morbidities	• Dementia • Stroke • Ischaemic Heart Disease • Diabetes Mellitus • Cancer • Congestive cardiac failure • Chronic Obstructive Pulmonary Disease • Parkinsonian syndromes • Hypertension • Depression	Multi-morbidity should be recorded as a count of morbidities present out of those specified. Individual morbidities should be recorded separately as binary variables. Specific morbidity guidance: Dementia – known diagnosis Stroke – any previous clinically symptomatic disease (not including transient ischaemic attacks) Cancer – active disease or treated disease within the last five years Congestive cardiac failure – symptomatic heart failure (e.g. requiring diuretic medication) of any cause, including heart failure with reduced ejection fraction and heart failure with preserved ejection fraction
Medication count	This should be recorded as a whole integer for total number of medications prescribed.	• All regular medication should be included regardless of compliance • Medications with multiple active ingredients should count as one drug (provided they are administered as a single tablet, inhalation etc) • As required medications should be included within count if prescribed • Inhalers, topical treatments, patches, and eye drops prescribed with therapeutic pharmacological intent should be included • Emollients and lubricating eye drops should not be included • Vitamins prescribed with therapeutic intent for deficiency replacement should be included (e.g. iron, vitamin B12) • Nutritional supplements (e.g. supplement drinks) should not be included • Over the counter medication taken for therapeutic intent (e.g. antihistamines) should be included
Functional ability	Basic ADLs: Barthel Index Instrumental ADLs: Nottingham Extended Activities of Daily Living	The score for each should be included within the dataset. Raw responses should be maintained locally. Nottingham EADLs specifically asks about activities actually conducted.
Frailty assessment	Clinical Frailty Scale (CFS)	Clinical Frailty Scale 2.0 (ordinal scale 1 to 9) – this should be assessed as part of a holistic assessment. For acute hospital admissions, this should be assessed considering their overall function/health two weeks prior to admission.
Cognition	Hospital setting: Screen for delirium using 4AT +/− DSM-5. Consider IQCODE Community setting: Prospective objective cognitive assessment	We do not make any specific recommendations on the tool to use for prospective cognitive assessment. Options may include: • MMSE • MoCA • Mini-ACE • ACE-III • Stroop test Record the outcome of the test (e.g. probable cognitive impairment vs no cognitive impairment), the raw total score, and assessment used.
Patient-reported outcome measures (PROMs)	We recommend that all studies should include some form of PROMs.	We make no specific recommendations on which tools to use. Options may include: • Eq. 5D • SF-36 • PROMIS Physical Function Short Form 10 • PROCOG

**Table 2:** Extended dataset criteria and guidance for scoring/completion

	Criteria	Guidance
Additional demographic data	• Self-declared disability • Religion • Biological sex • Highest education • Sexual orientation	We recommend following guidance set out by the Office for National Statistics in how these are categorised: https://www.ons.gov.uk/methodology/classificationsandstandards/measuringequality Note that religion may be recorded differently across devolved nations. The draft CRF includes all options to enable cross-nation applicability. The ONS (2004) definition of educational attainment is suggested for simplicity and applicability to an older population.
Additional multi-morbidity data	CIRS-G	Both individual scores for each body system and the overall total score should be recorded.
Handgrip strength	Record as whole number in kg.	Record the best measurement on each side (and best overall). The dominant side should be specified.
Walking speed	Record as m/s to two decimal places.	Specify course length used and whether start/stop were active walking.
Frailty phenotype	Record as overall score 0 to 5.	Specify how low physical activity has been defined.
Frailty Index	Record to two decimal places.	Suggest using deficits from previously validated indices e.g. eFI, ELSA, CSHA
Mood	GDS-15	Record total score.
Nutrition	Screening: MNA-SF Assessment: MNA (Full Form)	Record total score and code as “not malnourished”, “at risk of malnutrition”, or “malnourished” as per cut-offs.

### Core dataset

#### Demographics

Age, gender, ethnicity, socioeconomic status, and place of residence should be recorded for all patients. Ethnicity should be recorded as per UK census categories [11]. Within England, the Index of Multiple Deprivation decile should be calculated prospectively from postcode [12].

### Multiple long-term conditions

There is increasing evidence for multiple long-term conditions as a potential driver of poor health and frailty [13, 14] but there is no definitive preferred method of classification of multi-morbidity. Available methods, such as disease counts or co-morbidity indices, have limitations and fail to consider the complexities of accumulating multiple disease processes, all with different and potentially aggregated effects on an individual’s health [15, 16]. We have recorded long-term conditions as a count of the ten most highly rated conditions by the Delphi process. Each condition should be recorded separately as an individual binary variable.

### Medication count

We have also included a medication count. Guidance on what is considered a medication is shown in Table 1. The strict details of medications have not been included within the dataset for simplicity, but we do recommend that these are recorded and collected locally on CRFs. Dependent on the nature of the study, local CRFs may record drug names only, or may also record strength, dosing instructions, and indications.

### Functional ability

Functional dependence is an excellent marker of global health and fundamental in the assessment of frailty [17]. We have included a choice of assessment of functional independence depending on the research cohort being assessed. The Barthel Index [18] is recommended for assessment of basic Activities of Daily Living (ADLs) and the Nottingham Extended Activities of Daily Living (NEADLs) [19] is recommended for instrumental ADLs. The chief investigator should choose the most appropriate assessment for the cohort being assessed. Where possible, both tools should be used to avoid ceiling and floor effects. However, we recognise that assessment of instrumental ADLs may be less applicable to research in very dependent populations, such as residents in long-term 24-hour care. The NEADLs consists of many overlapping data variables and can be time-consuming to complete. NEADLs has been shown to have less sensitivity to change than quality of life measures in orthopaedic surgery populations; using this tool as a dynamic measure was not discussed within this consensus process [20]. The Disability Assessment of Dementia (DAD) [21] is recommended specifically for dementia populations, and can be recorded alongside the Barthel Index and NEADLs.

### Frailty assessment

Clinical Frailty Scale (CFS) should be included in all prospective geriatric medicine research studies. It is easy to assess with minimal training, has been validated in multiple settings, and is increasingly embedded into routine clinical care in the UK [22, 23]. Assessment of CFS can be conducted by all researchers, provided sufficient training and support is available [24]. Assessment of frailty by a Frailty Index and/or Fried phenotype are included within the extended dataset.

### Cognition

Assessment of cognition in some form is considered essential to all geriatric medicine research. However, how this is assessed will depend upon the population being examined. It was not possible to reach a consensus decision upon assessment of cognition appropriate for all populations. In hospital populations, all studies should incorporate delirium assessment. As a minimum, this should involve screening with the 4AT [25], and ideally full assessment according to the Diagnostic and Statistical Manual of Disorders 5 (DSM-5) [26]. The 4AT should be recorded separately where both screening and assessment are performed. In stable community settings, formal cognitive assessment should be performed using a recognised tool e.g. Stroop Test [27], Mini-Mental State Examination (MMSE) [28], Montreal Cognitive Assessment (MoCA) [29], Addenbrooke’s Cognitive Assessment III (ACE-III) [30], or Mini-ACE [31]. The Informant Questionnaire on Cognitive Decline (IQCODE) can be used to screen for pre-existent cognitive impairment when formal cognitive assessments are not appropriate, such as in patients with delirium [32].

### Patient-reported outcome measures

We also recommend that all prospective geriatric medicine research studies should incorporate some form of patient-reported outcome measures (PROMs). The tools that are used will be dependent on the research population and the research questions, but may include assessment of quality of life (e.g. EQ-5D [33] or Short Form 36, SF-36 [34]), perceived physical function (e.g. Patient Reported Outcome Measures Information System, PROMIS®, Physical Function Short Form 10 [35]), or cognitive function (e.g. Patient-Reported Outcomes in Cognitive Impairment, PROCOG [36]).

#### Extended dataset

##### Additional demographic data

Additional demographic data that may be required include data on disability (self-declared), religion, biological sex, household income, and sexual orientation. Within the UK, we suggest that these are coded to reflect the UK census categories [37]. Recording of this information is increasingly encouraged to ensure that research is representative.

##### Multiple long-term conditions

We would recommend Cumulative Illness Rating Scale-Geriatric (CIRS-G) if a rating scale of long-term conditions is required in addition to a count of diseases and medications. This was adapted from the original CIRS to reflect common problems in older adults with an emphasis on morbidity [38]. Rating scales of multi-morbidity do not reflect the interplay between the different morbidities and often ignore the importance of mental health [39]. CIRS-G does include a section on psychiatric illness but underplays the impact of dementia on an individual’s global health.

##### Handgrip strength

Use of a Jamar dynamometer is recommended for handgrip strength, as this device has been shown to have excellent concurrent validity with known weights [40]. However, other devices may be used if these have been fully calibrated (e.g. Takei Grip D). The device used should be specified on the dataset, as well as the date of last calibration. Yearly calibration of devices is recommended; this may need to be increased to six-monthly if devices are frequently transported in vehicles. Handgrip strength should be recorded on both sides, ideally with the participant sat out in a chair, with their elbow bent at 90^o^. If it is not possible for the participant to sit out due to fatigue, weakness, or illness effects, handgrip strength should be measured in the most upright position possible, and this should be recorded. The participant should be asked to “squeeze as hard as [they] can” [40]. We did not reach a consensus between two or three tries on each side although the commonly used Southampton protocol recommends three times on each side [40]. We recommend recording the number of attempts on the dataset. The best recording on each side, and best overall should be recorded on the dataset, alongside the participant’s hand dominance.

##### Walking speed

Usual gait speed should be measured by asking the participant to “walk at a normal comfortable pace”. Usual gait speed should be recorded as the course length (in metres) divided by the time (in seconds) to walk this course. Mobility aids may be used if necessary, and the use of these should be recorded. However, we recognise that different settings may necessitate some differences in how this is measured. A four metre course is conventional, but course lengths may vary from two to ten metres. The time may be measured with the participant starting from stationary, or whilst actively walking from one metre before course start. We recommend that the protocol used is documented on the dataset, so that researchers can exact caution when making comparisons across datasets.

##### Frailty phenotype

The Frailty Phenotype is frequently adapted from the original version due to problems sometimes encountered with the original assessments. To ensure standardisation, we recommend that most aspects are recorded as per the original study dataset [41]. Handgrip strength and gait speed should be recorded as per the previous sections. However, energy expenditure may be recorded using surrogate assessments tools for participant-reported physical activity (e.g. Frailty Intervention Trial definition [42], Survey of Health, Ageing, and Retirement in Europe – SHARE [43], Rapid Assessment of Physical Activity – RAPA [44], Physical Activity Scale for the Elderly – PASE [45]).

##### Frailty index

Frailty Index is a composite multi-dimensional score. Frailty Indices derived from different randomly selected variables (deficits) within the same population have been shown to closely correlate [46]. A Frailty Index should be calculated by dividing the number of deficits present by the total number of deficits measured. Measurement of between 30 and 50 variables is recommended. Frailty Indices should be recorded on the dataset, with reference to where information on the variables included can be found. When considering what variables to include within their index, researchers should consider selecting these from previously validated indices e.g. electronic Frailty Index (eFI) [47], English Longitudinal Study of Ageing [48], or original variables included in the Canadian Study of Health and Aging [23].

##### Mood

The Geriatric Depression Scale 15 (GDS-15) is recommended to screen for depressive symptoms [49]. GDS-15 focuses on functional and mood symptoms of depression rather than somatic features which can be misleading in older adults. It has been validated in inpatients, outpatients, and primary care [50].

##### Nutrition

The Mini-Nutritional Assessment (MNA®) Short Form is recommended for nutritional screening [51]. Where a more in-depth assessment is required, this can be expanded to the MNA Full Form. These tools, as well as being simple and easy to complete, are well validated, frequently used in research, and have excellent diagnostic accuracy [52–54].

## Discussion

Our core and extended datasets represent current consensus opinion of academic geriatric medicine clinicians across the UK. We consider the development and further use of these datasets will strengthen collaboration between researchers and academic institutions. Datasets can be utilised in all prospectively conducted research, including clinical trials, cross-sectional studies, and cohort studies. Standardisation of data collection in these studies will enable datasets to be combined with ease for individual patient data meta-analyses, or secondary data analyses for clinical questions considered at a later stage. This will minimise timescales across the research process, by preventing the need for design and conduct of further prospective studies where data is already available to answer clinical questions by pooling data from multiple studies. This will also inherently reduce the burden of participation on older adults.

Similarly, ensuring that this dataset is used to clinically phenotype in mechanistic studies involving the use of biological specimens will ensure discovery science findings have agreed clinical correlates, and facilitate collaboration and sharing of specimens across sites. Stored specimens can be transferred to a single site for experiments to be conducted, and shared clinical data available from the dataset can be used to provide phenotypic comparisons. The use of the dataset in clinical trials will enable the comparison of effectiveness between multiple different interventions from separate studies, which might not otherwise have been compared directly.

Importantly, the use of our dataset will assist in streamlining of the overall research process. Overall time for study set-up will be reduced, as protocol design should require less time to consider variables to measure, and CRFs can be quickly developed from our templates. This process can often be most challenging and time-consuming for less experienced (early career) researchers. Our dataset will ensure that the variables that have been considered most important and core to geriatric medicine research within the UK are not omitted. It should be noted that the datasets are not designed to be exclusive, but instead provide minimum standards. Researchers may choose to record any number of additional variables to cover aspects not included (e.g. sensory impairment, pain, muscle quantity/quality), or aspects included in more detail (e.g. food diaries and Subjective Global Assessment [55] for nutrition).

This dataset has been designed predominantly to consider measures that should be included within geriatric medicine research. However, we consider that this dataset can be utilised in non-clinical ageing research, including both biological and sociological gerontology studies. None of the measures included in either the core or extended datasets require specialist expertise to collect, and can be completed with minimal training. The dataset may also be utilised by other clinical specialties when conducting research involving older adults e.g. respiratory medicine, general practice. This will broaden the applicability of research and offer increased opportunities for multidisciplinary and multispecialty collaboration. Consensus for this dataset has been reached by involving UK geriatricians only, and this dataset may not be applicable internationally. However, we support the development of similar international datasets considering cultural differences, and encourage international collaboration on research studies. We consider our dataset to be complementary to other datasets such as the Canadian Frailty Network initiative [8] and the Royal College of Physicians National Hip Fracture Database [56], which centralises collection of routinely collected data. Our dataset specifically relates to prospectively conducted research. However, we would strongly support the development of electronic datasets for amalgamating routinely collected data in geriatric medicine. The Geriatric Medicine Research Collaborative [1, 3] have previously led such initiatives successfully on time-limited projects relating to delirium [57, 58] and Coronavirus 2019 (COVID-19) [59].

### Limitations

We recognise that there are a number of limitations associated with this dataset, and the process of producing it. Firstly, the initial literature review did not take a systematic approach. Although the questions in the first questionnaire were sufficiently broad to overcome this, and responders were able to suggest additional aspects, it is possible that other important aspects were not considered. Secondly, although nurses, allied health professionals, and pharmacists were invited to participate, responders were predominantly doctors. Thirdly, it was not possible to reach a consensus for all items, therefore at times we have taken a pragmatic approach (e.g. we do not make any specific recommendation of tool to be used for cognition but instead suggest options). Considering the limitations of the dataset itself, the total number of items included is small. Whilst this improves efficiency, this may reduce the representativeness of data that is collected and collated if only the core dataset is frequently utilised. The Geriatric Expert Group of the EMA recommends measurement of walking speed, and ideally Short Physical Performance Battery, for baseline characterisation of frailty in clinical trials involving older adults. However, our Delphi process did not reach a consensus that this should be considered core to all research studies. It should be noted that our core dataset is designed to be very broadly applicable to all geriatric medicine research including studies of virtual/remote design, observational studies, and studies in hospitalised patients or nursed in bed. In such studies, assessment of physical performance and handgrip strength may represent unique challenges, although we also acknowledge that handgrip strength and physical performance can be measured remotely, this may not be possible for all research studies. We emphasise that aspects of the extended dataset are strongly encouraged where study design allows, and our guidance on assessment of these will promote standardised approaches.

Fourth, the Delphi process did not explore the concepts of dynamic change, or suggested intervals for measurement. Fifth, we have designed this dataset with prospective research studies in mind, and it is unlikely to be applicable to retrospective research studies. The use of routinely collected clinical data for secondary research analysis is an ever-expanding field; a separate process that considers what data should be extracted, and how this should be conducted would complement this dataset. Sixth, we appreciate that this dataset may have limited permanency, and may need to be continuously updated as new assessment tools become available and more widely utilised (e.g. body-worn sensor data) [60]. We encourage body-worn sensor data within future studies, but at present do not make any recommendations as to a standardised technology and this was not one of the aspects suggested during the Delphi process. Lastly, this dataset was derived from expert opinion only; the dataset has not been specifically trialled to assess how this will improve research conduct. Unless the dataset is widely utilised by researchers within geriatric medicine, it is unlikely to provide significant impact. We plan to disseminate this dataset through available channels including the British Geriatrics Society, the Geriatric Medicine Research Collaborative [1, 3], and the NIHR Ageing Clinical Research Network. Where possible, we recommend citation of this manuscript where our dataset is used to enable utilisation to be tracked. Factor analyses may provide value in determining which specific variables consistently provide the most predictive value against outcomes of value to older people.

These datasets have been designed to be general for all geriatric medicine research. Importantly, we involved researchers with different subspecialty research expertise at each stage of the Delphi process. However, we recognise that further challenges with standardisation of data collection exist within subspecialty areas. We encourage the formation of subgroups to enable the standardisation of specific measures within specific research fields (e.g. sarcopenia, dementia, continence).

## Conclusion

Using a modified Delphi process, we have derived UK core and extended datasets for geriatric medicine research. The use of these datasets will enable sharing and collation of data across sites for individual patient data meta-analyses, and secondary research analyses. This will promote collaboration between UK academic institutions, and streamline processes in research design, particularly for early career researchers.

## Supplementary Information

**Additional file 1.** Members of the UK Geriatric Medicine Core Dataset Extended Working Group.

**Additional file 2.** Questionnaires used in Step 2, 3, and 4 of the Delphi study.

**Additional file 3.** This file demonstrates each of the different aspects that were included or not included at each stage of the Delphi study.

**Additional file 4.** Template case report form for the core dataset to be used and adapted for research studies.

**Additional file 5.** Template case report form for the extended dataset to be used and adapted for research studies.

## Data Availability

All data generated or analysed during this study are included in this published article.
