# Supplementary material for: Development of a UK core dataset for geriatric medicine research: a position statement and results from a Delphi consensus process
Source: BMC Geriatr. 2023 Mar 23;23:168. doi: 10.1186/s12877-023-03805-5 (PMC10035483; doi:10.1186/s12877-023-03805-5)
Supplement: Supplementary file 1 — Additional file 1. Members of the UK Geriatric Medicine Core Dataset Extended Working Group. [file 12877_2023_3805_MOESM1_ESM.docx]

# Development of a UK core dataset for geriatric medicine research: a position statement and results from a Delphi consensus process

Members of the UK Geriatric Medicine Core Dataset Extended Working Group:

| Name | Affiliation | Background |
| --- | --- | --- |
| Carly Welch | University of Birmingham | CW is now a Consultant Geriatrician and has successfully completed her PhD in 2022. Whilst undertaking this programme of work she was a Higher Specialist Trainee in Geriatric Medicine conducting clinical research during her PhD programme. |
| Daisy Wilson | University of Birmingham | DW is a Higher Specialist Trainee in Geriatric Medicine and Academic Clinical Lecturer. She completed her PhD in 2018. |
| Avan A Sayer | AGE Research Group, Newcastle University | AS is a Professor of Geriatric Medicine. She was appointed as an NIHR senior investigator in 2018. Her research interests include ageing, sarcopenia, and multimorbidity. |
| Miles D Witham | AGE Research Group, Newcastle University | MW is a Professor of Trials for Older People and the national specialty lead for the NIHR Ageing CRN. |
| Thomas A Jackson | University of Birmingham | TJ is an Associate Professor and Consultant Geriatrician. He completed his PhD in 2016 investigating cognitive disorders in the general hospital. |
| Raj Rajkumar | Brighton and Sussex Medical School | RR is a Professor and Chair of Geriatric and Stroke Medicine. He has been the Chief Investigator on multi-centre trials involving older adults. |
| Jugdeep Dhesi | Guy’s and St Thomas’ NHS Foundation Trust | JD is a Consultant Geriatrician and Honorary Professor. Her research interests include optimisation and treatment od older adults undergoing surgery, and clinical service development. |
| Mary Ni Lochlainn | King’s College London | MN is a higher specialist trainee in geriatric medicine currently undertaking a PhD. |
| Terry Aspray | Newcastle University | TA is an Honorary Clinical Senior Lecturer with 30 years of research experience. |
| Richard Dodds | Newcastle University | RD was an Intermediate Clinical Fellow and Honorary Consultant Geriatrician. He completed his PhD in 2015. |
| James Frith | Newcastle University | JF is a Clinical Senior Lecturer who completed his PhD in 2010. |
| Sarah Richardson | Newcastle University | SR is an Academic Clinical Lecturer and Higher Specialist Trainee in Geriatric Medicine. |
| Ellen Tullo | Newcastle University | ET is a lecturer in ageing and health education. |
| Alison Yarnall | Newcastle University | AY is an Intermediate Clinical Fellow and Honorary Consultant Geriatrician. |
| Richard Walker | Northumbria Healthcare NHS Foundation Trust | RW is a Consultant Physician, Honorary Professor of Ageing and International Health, and Director of Research & Development at his hospital trust. |
| Emma Cunningham | Queen’s University Belfast | EC is a Clinical Lecturer who was awarded her PhD in 2015. |
| Josephine Prynn | University College London | JP is a PhD student and Higher Specialist Trainee in Geriatric Medicine. |
| Harnish Patel | University Hospital Southampton NHS Foundation Trust | HP is a Consultant Physician and Honorary Senior Clinical Lecturer. |
| Divya Tiwari | University Hospitals Dorset NHS Foundation Trust | DT is a Consultant Geriatrician who has previously acted as a local Principal Investigator on multi-centre research grants, and has conducted systems-based research. |
| Stephen Makin | University of Aberdeen | SM is a Senior Lecturer and Consultant Geriatrician interested in rural medicine. |
| Phyo Myint | University of Aberdeen | PM holds the position of Clinical Chair in Old Age Medicine, who holds many national roles on committees. |
| Emily Henderson | University of Bristol | EH is an Associate Professor of Ageing and Movement Disorders and the Chair of the Research and Academic Development Committee within the British Geriatrics Society. |
| Victoria Keevil | University of Cambridge | VK is a Consultant Geriatrician and Senior Research Associate. |
| Katherine Walesby | University of Edinburgh | KW is a Clinical research fellow and Geriatric Medicine Registrar. |
| Louise Allan | University of Exeter | LM is a Professor of Geriatric Medicine who has previously received multiple grants for research in cognitive disorders with ageing. |
| Jane Masoli | University of Exeter | JM is an Academic Clinical Lecturer who was awarded her PhD in 2021. |
| Terry Quinn | University of Glasgow | TQ holds the post of Senior Clinical Lecturer and Honorary Consultant Physician in Stroke Medicine who holds multiple editorial board positions. |
| Andrew P Clegg | University of Leeds | AP is Professor of Geriatric Medicine, Honorary Consultant Geriatrician, and Associate Director for Health Data Research UK North. |
| Matthew Hale | University of Leeds | MH is a geriatric medicine registrar and early career researcher previously employed as an Academic Clinical Fellow. |
| Simon Conroy | University of Leicester | SC is an Honorary Professor of Geriatric Medicine with particular interests in health services research. |
| Joanne Taylor | University of Manchester | JT is a Higher Specialist Trainee in Geriatric Medicine currently completing her PhD. |
| John Gladman | University of Nottingham | JG is Professor in Medicine of Older People, with over 30 years of research experience. |
| Adam Gordon | University of Nottingham | AG is a Professor of Care of Older People, and the current President of the British Geriatrics Society. |
| Rowan Harwood | University of Nottingham | RH is a Professor of palliative and end-of-life care and Editor in Chief of Age and Ageing. |
| Natalie Cox | University of Southampton | NC is a research fellow and Higher Specialist Trainee in Geriatric Medicine. |
| Helen Roberts | University of Southampton | HC is a Professor of Medicine for Older People who has previously held multiple national roles on committees in ageing research. |

Ranking of morbidities to be included in “Top 10”

| **Morbidity variable** | **Mean ranking** | **Minimum ranking** | **Maximum ranking** |
| --- | --- | --- | --- |
| Dementia | 2 | 1 | 6 |
| Stroke | 5 | 1 | 12 |
| Ischaemic Heart Disease (including MI) | 5.875 | 1 | 30 |
| Diabetes Mellitus | 7 | 1 | 16 |
| Cancer | 8.1875 | 4 | 20 |
| Congestive cardiac failure | 8.8125 | 1 | 27 |
| Chronic Obstructive Pulmonary Disease | 9 | 2 | 28 |
| Parkinsonian syndromes | 11.1875 | 2 | 24 |
| Hypertension | 11.5625 | 2 | 24 |
| Depression | 12.625 | 2 | 27 |
| Chronic Kidney Disease | 12.9375 | 2 | 29 |
| Atrial Fibrillation | 14.3125 | 5 | 23 |
| Previous fragility fracture | 14.375 | 2 | 33 |
| Urinary incontinence | 15.9375 | 2 | 32 |
| Syncope | 16.4375 | 7 | 33 |
| Osteoporosis | 17.125 | 7 | 27 |
| Liver disease | 17.625 | 4 | 29 |
| Osteoarthritis | 17.9375 | 6 | 28 |
| Anxiety | 18.4375 | 3 | 34 |
| Myocardial infarction | 18.6875 | 2 | 34 |
| Anaemia | 19.625 | 8 | 32 |
| Peripheral vascular Disease | 20.3125 | 8 | 30 |
| Asthma | 21.5 | 9 | 33 |
| Inflammatory arthropathy | 22.75 | 14 | 34 |
| Cataracts | 24.375 | 15 | 34 |
| Peripheral neuropathy | 24.8125 | 17 | 31 |
| Connective tissue disease | 25.375 | 13 | 34 |
| Retinopathy | 25.75 | 16 | 34 |
| Other lung disease | 26 | 9 | 34 |
| Leukaemia | 26.375 | 10 | 34 |
| Thyroid disease | 26.375 | 9 | 33 |
| Urinary system disorders | 26.625 | 3 | 34 |
| Peptic ulcer disease | 27.5625 | 19 | 33 |
| Lymphoma | 28.3125 | 15 | 34 |
