# Supplementary material for: Development of a UK core dataset for geriatric medicine research: a position statement and results from a Delphi consensus process
Source: BMC Geriatr. 2023 Mar 23;23:168. doi: 10.1186/s12877-023-03805-5 (PMC10035483; doi:10.1186/s12877-023-03805-5)
Supplement: Supplementary file 3 — Additional file 3. This file demonstrates each of the different aspects that were included or not included at each stage of the Delphi study. [file 12877_2023_3805_MOESM3_ESM.pdf]

	Step 1	Step 2	Step 3	Step 4	Step 5	Step 6	Final consensus
Demographics	Age<br>Gender<br>Ethnicity<br>Level of education<br>Social deprivation index<br>Smoking status<br>Alcohol use	Demographics - broad questioning	Age<br>Gender<br>Ethnicity<br>Level of education<br>Previous occupation<br>Nationality<br>Marital status<br>Social deprivation index<br>Postcode<br>Number of cars available to household<br>Age left formal education<br>Socioeconomic status<br>Biological sex<br>Smoking status<br>Alcohol use	Age - Agree as core?<br>Gender - Agree as core?<br>Ethnicity - Agree as core?<br>Level of education - extended?<br>Previous occupation - extended?<br>Nationality - extended?<br>Marital status - extended?<br>Social deprivation index - extended?<br>Postcode - extended?<br>Smoking status - extended?<br>Alcohol use - extended?	Age<br>Gender<br>Ethnicity<br>Social deprivation index discussed and agreed should be included as core<br>Discussed as part of personal characteristics - some funders (e.g. NIHR) now recommending collection of personal characteristics for all research. This may also include information of perceived personal disability, household income, and sexual identity. This information is important to collect to ensure research is representative, however, was not considered specifically core to this geriatric medicine dataset.		**Age**<br>**Gender**<br>**Ethnicity**<br>**Social deprivation index**
Morbidity	Free text<br>Limitations recognised from traditional disease counts or co-morbidity indices.<br>Dichotomously record morbidity included within frailty index	Morbidity - broad questioning	Include as core?<br>Free text or count/scale: Free text only<br>Free text and count/scale<br>Count/scale only<br>Morbidity scale: UK Biobank hierarchy of conditions<br>Charlson comorbidity index<br>Geriatric index of comorbidity<br>Specific diagnostic ICD-10 codes<br>Multimorbidity count<br>Binary "yes/no" for long-term conditions and multimorbidity count<br>Cumulative illness rating scale<br>Embedded within frailty index	Morbidity - agree as core?<br>Ranking of options<br>Free text only<br>Charlson comorbidity index<br>Free text and Charlson comorbidity index<br>Comorbidity count<br>Free text and comorbidity count	Multi-morbidity was discussed in depth at this meeting. It was considered that the previous rounds of the Delphi had led to a narrowing towards tools that may now be less relevant to complex geriatric medicine populations. Upon careful discussion and consideration, it was decided to conduct a further survey to identify "Top 10" long-term conditions that should be recorded as binary variables as part of geriatric medicine research.	Dementia<br>Stroke<br>Ischaemic Heart Disease (including MI)<br>Diabetes Mellitus<br>Cancer<br>Congestive cardiac failure<br>Chronic Obstructive Pulmonary Disease<br>Parkinsonian syndromes<br>Hypertension<br>Depression<br>Chronic Kidney Disease<br>Atrial Fibrillation<br>Previous fragility fracture<br>Urinary incontinence<br>Syncope<br>Osteoporosis<br>Liver disease<br>Osteoarthritis<br>Anxiety<br>Myocardial infarction<br>Anaemia<br>Peripheral vascular Disease<br>Asthma<br>Inflammatory arthropathy<br>Cataracts<br>Peripheral neuropathy<br>Connective tissue disease<br>Retinopathy<br>Other lung disease<br>Leukaemia<br>Thyroid disease<br>Urinary system disorders<br>Peptic ulcer disease<br>Lymphoma	**Dementia**<br>**Stroke**<br>**Ischaemic Heart Disease (including MI)**<br>**Diabetes Mellitus**<br>**Cancer**<br>**Congestive cardiac failure**<br>**Chronic Obstructive Pulmonary Disease**<br>**Parkinsonian syndromes**<br>**Hypertension**<br>**Depression**
Functional ability	Katz ADLs<br>Lawton ADLs	Functional ability - broad questioning	Include as core?<br>Basic activities of daily living (ADLs): Barthel index<br>Katz ADLs<br>As per English Longitudinal Study of Ageing<br>Office of Population Censuses and Surveys for disability<br>Instrumental activities of daily living (ADLs): Nottingham extended ADLs<br>Lawton ADLs<br>As per English Longitudinal Study of Ageing	Functional ability - agree as core?<br>Basic ADLs: Barthel index - agree?<br>Instrumental ADLs: Nottingham extended ADLs - agree?	Discussion that functional ability should always be recorded, but the inclusion of basic vs instrumental ADLs vs both would be dependent on the patient population<br>Basic ADLs: Barthel index<br>Disability Assessment in Dementia also encouraged in studies recruting patients with dementia specifically.<br>Instrumental ADLs: Nottingham extended ADLs		**Barthel index and/or Nottingham Extended Activities of Daily Living**
Nutrition		Nutrition - broad questioing	Include as core?<br>Risk of malnutrition: Malnutrition Universal Screening Tool (MUST)<br>Mini-Nutritional Assessment (Short Form)<br>Weight and Body Mass Index alone<br>Seniors in the community: risk evaluation for eating and nutrition, Version II<br>Short Nutritional Assessment Questionnaire<br>Full nutritional assessment: Mini-Nutritional Assessment (Full Form)<br>Subjective Global Assessment	Nutrition - extended?<br>MUST<br>MNA (Short Form)<br>MNA (Full Form) - agree?	Consensus that nutrition should be included in extended rather than core dataset. Agreement to use of MNA (Short Form) for screening, with extension to MNA (Full Form) would be pragmatic and enable ease of data synthesis across studies.		**MNA (Short form) for screening**<br>**MNA (Full form) for assessment**
Frailty	Fried frailty phenotype<br>Suggest record physical activity as per SHARE protocol.<br>Frailty index (FI)<br>Can use any variables, but potentially standardised variables proposed.<br>Clinical Frailty Scale (CFS)	Measures<br>Energy expenditure/ physical activity within Fried<br>Variables to include within standardised FI	Include as core?<br>Measures: Fried frailty phenotype<br>Frailty index (FI)<br>Clinical Frailty Scale (CFS)<br>PRISMA 7 questionnaire<br>Edmonton frail scale<br>Energy expenditure/ physical activity within Fried: As per Frailty Intervention Trial<br>Rapid Assessment of Physical Activity<br>As per SHARE protocol<br>Physical Activity Scale for the Elderly<br>Preference for standardised FI: As per electronic frailty index<br>As per English Longitudinal Study of Ageing<br>As per original Canadian Study of Health and Aging	Frailty - agree as core?<br>Ranking of options<br>Fried frailty phenotype only<br>Fried frailty phenotype and CFS<br>Fried frailty phenotype and FI<br>Fried phenotype, CFS, and FI<br>Frailty index only<br>FI and CFS<br>CFS only	Fried frailty phenotype included as part of extended dataset. No consensus reached on definition to use for physical activity, which should be recorded as part of the study.<br>Decision made to include this as part of an extended dataset. A lot of discussion about the variables that could be included within such an index. Agreed that any variables/ model may be used with recording of the FI and details of the model used would be pragmatic.<br>Consensus to include recording of CFS as part of all prospective geriatric medicine research.		**Fried: record overall score 0 to 5 and specify how physical activity recorded.**<br>**FI: record to two decimal places. Suggest using previously validated deficits.**<br>**CFS: assessed as part of holistic assessment.**
Body morphology	Body Mass Index<br>Waist:hip circumference<br>Weight loss indicating change in physical state	Body morphology	Include as core?<br>Height<br>Weight<br>Body Mass Index<br>Waist: hip circumference	Body morpholody - extended?			
Handgrip strength	Handgrip strength	Handgrip strength - broad questioning	Include as core?<br>Jamar: Should all research use Jamar? Yes/No<br>Protocol: Best of three - dominant hand only<br>Two readings on both sides - best of all readings<br>Best of two - dominant side only<br>Three readings on each side - best of all readings<br>Measure dominant and non-dominant readings and take the average of multiple readings on each side	Handgrip strength - extended?<br>Dominant side only<br>Both sides<br>2 each side<br>3 each side	Agreed to include as part of extended dataset. Record the best measurement on each side and best overall. Dominant side should be recorded.		**Record as whole number in kg - best overall and on each side.**
Usual gait speed	Usual gait speed	Usual gait speed - broad questioning	Include as core?<br>Distance of course: 3m<br>4m<br>5m<br>10m<br>"Start" and "stop" whilst active walking: Active walking? Yes/No	Usual gait speed - extended?<br>Ranking of options<br>3m from stationary<br>3m with 1m active walking<br>4m from stationary<br>4m with 1m active walking<br>5m from stationary<br>5m with 1m active walking<br>10m from stationary<br>10m with 1m active walking	Included as part of extended dataset. Unable to reach consensus on course length or active walking. Course length and whether start/stop were active walking should be recorded.		**Record as m/s to two decimal places and course length used.**
Additional physical performance			Usual gait speed only<br>Short physcial performance battery (including usual gait speed)<br>6-minute walk distance<br>Timed up and go<br>Chair stands only				
Cognition	Considered MoCA may be associated with cognitive burden.<br>Abbreviated Mental Test Score (10 point)<br>Addenbrooke's Cognitive Examination III (ACE III) considered overly burdensome for many research studies, but could be included as part of an extended dataset.<br>Stroop test as alternative to AMTS or within extended dataset (recognition that copyright is limitation). Takes only four minutes with minimal burden to participants.	Cogntion - broad questioning	Include as core?<br>Montreal Cognitive Assessment (MoCA)<br>Abbreviated Mental Test Score (10 point)<br>Mini-Mental State Examination<br>Addenbrooke's Cognitive Examination III (ACE III)<br>Stroop test<br>Abbreviated Mental Test Score (4 point) - as part of 4AT<br>Informant Questionnaire on Cognitive Decline in the Elderly (Short Form)<br>Colour trails<br>Fluency and clock drawing taken from ACE III (potential to expand to full ACE III)	Cognition - agree as core?<br>MoCA - agree to use?	This was strongly considered to be core to geriatric medicine research by many who attended the consensus meeting. However, it was considered that the earlier removal of delirium didn't fully appreciate how research may need to be adapted dependent on setting. Agreed screening for delirium (4AT +/-DSM-5) should be performed in research in hospital settings, whereas prospective objective cognitive assessment should be performed in community settings. We were unable to reach consensus on the specific tools to be used for cognitive assessment, but all those that were discussed have been included as possible options.		**Hospital setting:**<br>**Screen for delirium (4AT +/- DSM-5)**<br>**Community setting:**<br>**Prospective cognitive assessment. Record score and tool used.**
Mood	GDS-4 has been shown to have similar clinical utility to longer versions (GDS-5, GDS-15, GDS-30).	Mood - broad questioning	Include as core?<br>Hospital Anxiety and Depression Scale<br>Geriatric Depression Scale (GDS) -1<br>GDS-4<br>GDS-5<br>GDS-15<br>Simple questioning: is there a history of a mood disorder?	Mood - extended?<br>GDS-15 - agree?	GDS-15 included within extended dataset		**Record total GDS-15 score**
Delirium	Screening for delirium using 4AT in patients who are acutely unwell.	Delirium - broad questioning	Agree as core?<br>4AT<br>4AT then DSM-5<br>SqID<br>SqID then DSM-5<br>DSM-5 alone (no need for screening tool)<br>Confusion Assessment Method	Delirium - extended?<br>4AT - agree?	See cognition section above		
Storing biological specimens	Store serum and plasma for all participants<br>Where possible, also store peripheral blood mononuclear cells as well as serum/plasma	Storing biological specimens - broad questioning	Include as core?<br>Store in a link anonymised manner linked to core dataset<br>Freeze at -20°C (or lower)<br>Freeze at -70°C<br>Freeze at -80°C<br>Ethics to allow samples to be moved to other centres<br>Storage of peripheral blood mononuclear cells as well as serum/plasma<br>Storage of some specimens in plates and some in cryovials				
Muscle quantity/ quality	Extended dataset<br>Ultrasound - Bilateral Anterior Thigh Thickness as per Wilson et al<br>Ultrasound recognised to be more widely available and applicable across settings than DXA or MRI	Muscle quantity/ quality - broad questioning	Include as core?<br>Ultrasound - Bilateral Anterior Thigh Thickness as per Wilson et al<br>Ultrasound - Rectus Femoris cross-sectional area<br>Bioelectrical Impedance Analysis<br>Impedance and reactance recorded alongside height, weight, and gender, as well as any specific results dependent on the device and protocol<br>Dual energy X-ray Absorptiometry (DXA)<br>Magnetic Resonance Imaging (MRI)<br>Computed Tomography (CT)				
Quality of life			Quality of life - broad questioning	Quality of life - extended?<br>EQ5D<br>SF36	Decision that all research should include prospectively recorded patient-reported outcomes, which may include quality of life, but could include e.g. perceived physical function.		**No specific recommendation on tool to be used, but recognition that all prospective research should include PROMs.**
Medication count			Medication count - broad questioning	Medication count - agree as core?	Discussed what should be considered within medication count. Consensus decision was that this should be included with guidance as to what to include.		**Medication count - drugs used to be recorded locally**
Behavioural and Psychotic Symptoms			Behavioural and Psychotic Symptoms - broad questioning				
Pain			Pain - broad questioning				
Data from caregivers			Data from caregivers - broad questioning				
Place of residence			Place of residence - include as core?	Place of residence - agree as core?	Core dataset. Minimum information required: independent living/ additional care/ residential care home/ nursing home		**Place of residence within demographics.**
Walking aids used			Walking aids used - include as core?	Walking aids used - extended?			
Visual and hearing impairment			Visual and hearing impairment - include as core?	Visual and hearing impairment - extended?			
Falls over last year			Falls over last year - include as core?	Falls over last year - extended?			
Continence			Continence - broad questioning	Continence - extended?<br>Recording as part of Barthel index sufficient - agree?	Recording as part of Barthel index		
Admissions over last year			Admissions over last year - include as core?	Admissions over last year - extended?			
