# Supplementary material for: Development of a UK core dataset for geriatric medicine research: a position statement and results from a Delphi consensus process
Source: BMC Geriatr. 2023 Mar 23;23:168. doi: 10.1186/s12877-023-03805-5 (PMC10035483; doi:10.1186/s12877-023-03805-5)
Supplement: Supplementary file 4 — Additional file 4. Template case report form for the core dataset to be used and adapted for research studies. [file 12877_2023_3805_MOESM4_ESM.pdf]

Template case report form – Geriatric medicine core dataset V1.0

Demographics

<table>
<tr><td>Age</td><td colspan="4">___ ___ ____</td></tr>
<tr><td>Gender (circle)</td><td colspan="2">Male</td><td colspan="2">Female</td></tr>
<tr><td rowspan="6">Ethnicity (circle)</td><td>White British</td><td>White Irish</td><td colspan="2">White Other</td></tr>
<tr><td>White & Black Caribbean</td><td>White & Black African</td><td>White & Asian</td><td>Other mixed background</td></tr>
<tr><td>Indian</td><td>Bangladeshi</td><td>Pakistani</td><td>Other Asian background</td></tr>
<tr><td>Caribbean</td><td>African</td><td colspan="2">Black other</td></tr>
<tr><td>Chinese</td><td colspan="3">Other (specify)</td></tr>
<tr><td>Place of residence (circle)</td><td>Independent living</td><td>Additional care</td><td>Residential care home</td><td>Nursing home</td></tr>
<tr><td>Socioeconomic decile (calculate for postcode)</td><td colspan="4"></td></tr>
</table>

Multi-morbidity

IHD including MI	Y/N	Congestive cardiac failure	Y/N
Dementia	Y/N	COPD	Y/N
Stroke	Y/N	Parkinsonian syndrome	Y/N
Diabetes	Y/N	Hypertension	Y/N
Active cancer	Y/N	Depression	Y/N

Count _______

Medications

Please list all medications below:

Medication Count _______

Barthel Index

Please circle the most appropriate responses

	0	1	2	3
Bowels	Incontinent	Occasional accident	Continent	
Bladder	Incontinent (or catheter unable to manage independently)	Occasional accident (max once per 24 hours)	Continent (over 7-day period)	
Grooming	Needs help with personal care	Independent face/hair/teeth/shaving		
Toilet Use	Dependent	Needs some help	Independent	
Feeding	Unable	Needs help e.g. cutting	Independent	
Transfers	Unable – no sitting balance	Major help but can sit	Minor help (physical or verbal)	Independent
Mobility	Immobile	Wheelchair independent	Walks with help of one person	Independent (may use aids)
Dressing	Dependent	Needs help, can do about half	Independent	
Stairs	Unable	Needs help	Independent	
Bathing	Dependent	Independent		

Barthel Index Score _________

Nottingham Extended Activities of Daily Living

This should be completed according to the activities that the participant has actually done over the last four weeks. Please tick the most appropriate responses.

	0		1	
	Not at all	With help	On own with difficulty	On own
Walk around outside?				
Climb stairs?				
Get in and out of car?				
Walk over uneven ground?				
Cross roads?				
Travel on public transport?				
Manage to feed yourself?				
Make a hot drink?				
Take hot drinks from one room to another?				
Do the washing up?				
Make a hot snack?				
Manage your own money?				
Wash small items of clothing?				
Do your own housework?				
Do your own shopping?				
Do a full clothes wash?				
Read newspapers or books?				
Use the telephone?				
Write letters? *(or emails)*				
Go out socially?				
Manage your own garden?				
Drive a car?				

NEADL Score _______

Template case report form – Geriatric medicine core dataset V1.0

Cognition

Assessment Used __________________________ Score _______

Clinical Frailty Scale

Level of fitness/ frailty	Description	Number (circle best fitting level)
Very fit	Robust, active, energetic and motivated; commonly exercise regularly	1
Well	No active disease symptoms but less fit than above; exercise often or active occasionally e.g. seasonally	2
Managing well	Medical problems are well controlled but not regularly active beyond routine walking	3
Living with very mild frailty	Not dependent on others for daily help but often symptoms limit activities; common complaint is being “slowed-up” or tired during the day	4
Living with mild frailty	More evident slowing; need help with high order IADLs; typically progressively impairs shopping and walking outside alone, meal preparation and housework	5
Living with moderate frailty	Need help with all outside activities; often have problems with stairs and need help with bathing and might need minimal assistance with dressing	6
Living with severe frailty	Completely dependent for personal care; seem stable and not at high risk of dying within 6 months	7
Living with very severe frailty	Completely dependent, approaching the end of life; typically, could not recover from a minor illness	8
Terminally ill	Life expectancy < 6 months but not otherwise evidently frail	9
