# Supplementary material for: Development of a UK core dataset for geriatric medicine research: a position statement and results from a Delphi consensus process
Source: BMC Geriatr. 2023 Mar 23;23:168. doi: 10.1186/s12877-023-03805-5 (PMC10035483; doi:10.1186/s12877-023-03805-5)
Supplement: Supplementary file 5 — Additional file 5. Template case report form for the extended dataset to be used and adapted for research studies. [file 12877_2023_3805_MOESM5_ESM.pdf]

Template case report form – Geriatric medicine extended dataset V1.0

Demographics

<table>
<tr><td>Biological sex (circle)</td><td colspan="2">Male</td><td colspan="2">Female</td></tr>
<tr><td rowspan="6">Religion (circle)</td><td>No religion</td><td colspan="2">Catholic</td><td>Presbyterian</td></tr>
<tr><td>Church of England</td><td>Church of Scotland</td><td>Church of Ireland</td><td>Free presbyterian</td></tr>
<tr><td>Methodist</td><td>Baptist</td><td>Protestant – Other</td><td>Christian – Other</td></tr>
<tr><td>Buddhist</td><td colspan="2">Hindu</td><td>Jewish</td></tr>
<tr><td>Muslim</td><td colspan="2">Sikh</td><td>Other</td></tr>
<tr><td colspan="4">Prefer not to say</td></tr>
<tr><td rowspan="3">Highest education (circle)</td><td>Higher education and professional/vocational equivalents</td><td>A levels, vocational level 3, and equivalents</td><td colspan="2">GCSE/O level grade A*-C, vocational level 2 and equivalents</td></tr>
<tr><td>Qualifications at level 1 and below</td><td>Other qualifications: level unknown (including international)</td><td colspan="2">No qualifications</td></tr>
<tr><td colspan="4">Prefer not to say</td></tr>
<tr><td rowspan="2">Sexual orientation (circle)</td><td>Heterosexual (straight)</td><td>Gay/ Lesbian</td><td>Bisexual</td><td>Other</td></tr>
<tr><td colspan="4">Prefer not to say</td></tr>
<tr><td rowspan="2">Self-declared disability (circle)</td><td>None</td><td>Physical disability</td><td>Cognitive disability</td><td>Physical and cognitive disability</td></tr>
<tr><td colspan="4">Prefer not to say</td></tr>
</table>

Cumulative Illness Rating Scale – Geriatric (CIRS-G)

Heart	No problem	0 ☐
	MI >5 years ago, occasional angina treated with meds as needed	+1 ☐
	CHF compensated with meds, daily antianginal meds, left ventricular hypertrophy, atrial fibrillation, bundle branch block, daily antiarrhythmic drugs	+2 ☐
	MI ≤5 years ago, abnormal stress test, or past PTCA or CABG	+3 ☐
	Marked activity restriction secondary to cardiac status (i.e., unstable angina or intractable CHF)	+4 ☐
Vascular	No problem	0 ☐
	Hypertension compensated with salt restriction and weight loss, cholesterol >200 mg/dL	+1 ☐
	Daily antihypertensive meds, one symptom of atherosclerotic disease (angina, claudication, bruit, amaurosis fugax, absent pedal pulses), aortic aneurysm <4 cm	+2 ☐
	≥2 symptoms of atherosclerosis	+3 ☐
	Previous vascular surgery, aortic aneurysm ≥4 cm	+4 ☐
Hematopoietic	No problem	0 ☐
	Hemoglobin: females 10-12 g/dL, males 12-14 g/dL, anemia of chronic disease	+1 ☐

	Hemoglobin: females 8 to <10 g/dL, males 10 to <12 g/dL, anemia secondary to iron/vitamin B-12/folate deficiency or chronic renal failure, total WBC 2,000-4,000	+2 ☐
	Hemoglobin: females <8 g/dL, males <10 g/dL, total WBC <2,000	+3 ☐
	Any leukemia or lymphoma	+4 ☐
Respiratory	No problem	0 ☐
	Recurrent episodes of acute bronchitis, current treated asthma with inhalers as needed, cigarette smoker 10-20 pack years	+1 ☐
	X-ray evidence of COPD, requires daily theophylline or inhalers, treated for pneumonia two or more times in the past 5 years, smoked 21-40 pack years	+2 ☐
	Limited ambulation secondary to limited respiratory capacity, requires oral steroids for lung disease, smoked >40 pack years	+3 ☐
	Requires supplemental oxygen, ≥1 episode of respiratory failure requiring assisted ventilation, any lung cancer	+4 ☐
Eyes, ears, nose, throat, and larynx	No problem	0 ☐
	Corrected vision 20/40, chronic sinusitis, mild hearing loss	+1 ☐
	Corrected vision 20/60 or reads newsprint with difficulty, requires hearing aid, chronic sinonasal complaints requiring medication, requires medication for vertigo	+2 ☐
	Partially blind (requires an escort to venture out), unable to read newsprint, conversational hearing still impaired with hearing aid	+3 ☐
	Functional blindness, functional deafness, laryngectomy, requires surgical intervention for vertigo	+4 ☐
Upper GI	No problem	0 ☐
	Hiatal hernia, heartburn complaints treated with as-needed meds	+1 ☐
	Needs daily H_2 blocker or antacid, documented gastric or duodenal ulcer within 5 years	+2 ☐
	Active ulcer, guaiac positive stools, any swallowing disorder or dysphagia	+3 ☐
	Gastric cancer, history of perforated ulcer, melena or hematochezia from upper GI source	+4 ☐
Lower GI	No problem	0 ☐
	Constipation managed with meds as needed, active hemorrhoids, status post hernia repair	+1 ☐
	Requires daily bulk laxatives or stool softeners, diverticulosis, untreated hernia	+2 ☐
	Bowel impaction in the past year, daily use of stimulant laxatives or enemas	+3 ☐
	Hematochezia from lower GI source, currently impacted, diverticulitis flare up, status post bowel obstruction, bowel carcinoma	+4 ☐
Liver, pancreas, and biliary	No problem	0 ☐
	History of hepatitis >5 years ago, cholecystectomy	+1 ☐
	Mildly elevated LFTs (≤150% of normal), hepatitis within 5 years, cholelithiasis, daily or heavy alcohol use within 5 years	+2 ☐

	Elevated bilirubin (total >2 mg/dL), marked elevation of LFTs (>150% of normal), requires supplemental pancreatic enzymes for digestion	+3 ☐
	Clinical or lab evidence of biliary obstruction, any biliary tree carcinoma, cholecystitis, pancreatitis, active hepatitis	+4 ☐
Renal	No problem	0 ☐
	Kidney stone passage within the past 10 years or asymptomatic kidney stone, pyelonephritis within 5 years	+1 ☐
	Serum creatinine 1.5-3.0 mg/dL without diuretic or antihypertensive medication	+2 ☐
	Serum creatinine >3.0 mg/dL OR serum creatinine >1.5 mg/dL on diuretic, antihypertensive, or bicarbonate therapy, current pyelonephritis	+3 ☐
	Requires dialysis, renal carcinoma	+4 ☐
Genitourinary	No problem	0 ☐
	Stress incontinence, hysterectomy, BPH without urinary symptoms	+1 ☐
	Abnormal pap smear, frequent UTIs (≥3 in past year), urinary incontinence (non-stress) in females, BPH with hesitancy or frequency, current UTI, any urinary diversion procedure, status post TURP	+2 ☐
	Prostate cancer in situ (i.e., found incidentally during TURP), vaginal bleeding, cervical carcinoma in situ, hematuria, status post urosepsis in past year	+3 ☐
	Acute urinary retention, any GU carcinoma except as above	+4 ☐
Musculoskeletal and skin	No problem	0 ☐
	Uses meds as needed for arthritis or has mildly limited activities of daily living (ADLs) from joint pathology, excised non-melanoma skin cancers, skin infections requiring antibiotics within a year	+1 ☐
	Daily antiarthritic meds or use of assistive devices or moderate limitation in ADLs, daily meds for chronic skin conditions, melanoma without metastasis	+2 ☐
	Severely impaired ADLs secondary to arthritis, requires steroids for arthritic condition, vertebral compression fractures from osteoporosis	+3 ☐
	Wheelchair bound, severe joint deformity or severely impaired usage, osteomyelitis, any bone or muscle carcinoma, metastatic melanoma	+4 ☐
Neurologic	No problem	0 ☐
	Frequent headaches requiring meds as needed without interference with daily activities, history of TIA phenomena (at least one)	+1 ☐
	Requires daily meds for chronic headaches or headaches that regularly interfere with daily activities, status post CVA without significant residual, mild neurodegenerative disease (Parkinson's, MS, ALS, etc)	+2 ☐
	Status post CVA with mild residual dysfunction, any CNS neurosurgical procedure, moderate neurodegenerative disease	+3 ☐
	Status post CVA with mild residual dysfunction, any CNS neurosurgical procedure, moderate neurodegenerative disease	+4 ☐
Endocrine and breast	No problem	0 ☐
	Diabetes mellitus compensated with diet, obesity (BMI >30), requires thyroid hormone replacement	+1 ☐

	Diabetes mellitus requiring insulin or oral agents, fibrocystic breast disease	+2 ☐
	Any electrolyte disturbance requiring hospital treatment, morbid obesity (BMI >45)	+3 ☐
	Brittle or poorly controlled diabetes mellitus or diabetic coma in the past year, requires adrenal hormone replacement, adrenal, thyroid, or breast carcinoma	+4 ☐
Psychiatric illness	No problem	0 ☐
	Minor psychiatric condition or history thereof: specifically, previous outpatient mental health treatment during a crisis, outpatient treatment for depression >10 years ago, current use of minor tranquilizers for episodic anxiety (occasional usage), mild early dementia	+1 ☐
	A history of major depression (by DSM) within the past 10 years (treated or untreated), mild dementia, any previous psychiatric hospitalization, any psychotic episode substance abuse history >10 years ago	+2 ☐
	Currently meets DSM criteria for major depression or two or more episodes of major depression in the past 10 years, moderate dementia, current usage of daily anti-anxiety medication, currently meets DSM criteria for substance abuse or dependency, requires daily antipsychotic medication	+3 ☐
	Current mental illness requiring psychiatric hospitalization, institutionalization, or intensive outpatient management (e.g. patients with severe or suicidal depression, acute psychosis or psychotic decompensation, severe agitation from dementia, severe substance abuse, etc), severe dementia	+4 ☐

Total CIRS-G Score ☐☐

Handgrip strength

Device used: ________________________________ Calibration date: ____________________

Position measurement performed (circle): Sat in chair/ Semi-upright on bed or couch

Dominant arm (circle): Right/ Left

Right	1.	Best
	2.	
	3.	
Left	1.	Best
	2.	
	3.	

Walking speed

Course length: __________

Active walking for start: Yes/ No Active walking for stop: Yes/ No

Time taken: _____:______ (sec: millisec)

Frailty Phenotype

Adapt according to research design and tool used to assess physical activity. The example given is taken from the Frailty Intervention Trial, and may be adjusted accordingly. Handgrip strength and walking speed should be recorded separately above, and the measures used to calculate the score below.

Shrinking (Score 1 if either yes)	4.5kg weight loss over last year?		Yes	No
	Over 5% loss of previous year's body weight on examination with scales		Yes	No
Weakness – handgrip strength (score 1 if below or equal to cut-offs)	BMI – Male	Cutoff (kg)	BMI - Female	Cutoff (kg)
	<=24	<=29	<=24	<=17
	24-26	<=30	24-26	<=17.3
	26-28	<=30	26-28	<=18
	>28	<=32	>28	<=21
Self-reported exhaustion (score 1 if answers most or all of the time to either question)	How often over the last week have you felt that the following statements were true:			
	I felt that everything I did was an effort		None of the time	
			Some of the time	
			Most of the time	
			All of the time	
	I could not get going		None of the time	
			Some of the time	
			Most of the time	
			All of the time	
Gait speed (score 1 if below or equal to cut-offs)	Height (cm) – Male	Cutoff (m/s)	Height (cm) – Female	Cutoff (m/s)
	<=173	<=0.65	<=159	<=0.65
	>173	<=0.76	>159	<=0.76
Low physical activity (score 1 if answers yes to all three questions)	In the last three months, have you:			
	Performed no weight-bearing physical activity		Yes	No
	Spent more than 4 hours/ day sitting		Yes	No
	Been for a short walk once/ month or less frequently		Yes	No

Total score: /5

Frailty Index

If adapted from a validated index, please specify which: ____________________

Frailty index: __________________

Geriatric Depression Scale – 15 (GDS-15)

Please ask the participants the following questions, and ask them to answer yes or know to each question. Please circle their responses.

1) Are you basically satisfied with your life?	Yes<br>No (+1)
2) Have you dropped many of your activities and interests?	Yes (+1)<br>No
3) Do you feel that your life is empty?	Yes (+1)<br>No
4) Do you often get bored?	Yes (+1)<br>No
5) Are you in good spirits most of the time?	Yes<br>No (+1)
6) Are you afraid that something bad is going to happen to you?	Yes (+1)<br>No
7) Do you feel happy most of the time?	Yes<br>No (+1)
8) Do you often feel helpless?	Yes (+1)<br>No
9) Do you prefer to stay at home, rather than go out and do new things?	Yes (+1)<br>No
10) Do you feel you have more problems with memory than most?	Yes (+1)<br>No
11) Do you think it is wonderful to be alive?	Yes<br>No (+1)
12) Do you feel pretty worthless the way you are now?	Yes (+1)<br>No
13) Do you feel full of energy?	Yes<br>No (+1)
14) Do you feel that your situation is hopeless?	Yes (+1)<br>No
15) Do you think that most people are better off than you are?	Yes (+1)<br>No

Total score: /15

0-4: Normal 5-9: Mild depressive symptoms 10-15: More severe depressive symptoms

Mini-Nutritional Assessment

Circle the most appropriate responses. If only screening to be performed, only white sections need be included. To extend to full assessment grey sections should be included.

	Options		Points
Has food intake declined over the past 3 months due to loss of appetite, digestive problems, chewing or swallowing difficulties?	Severe decrease		0
	Moderate decrease		1
	No decrease		2
Weight loss during the last 3 months	> 3kg		0
	Does not know		1
	1-3kg		2
	No weight loss		3
Mobility	Bed or chair bound		0
	Able to get out of bed/ chair but does not go out		1
	Goes out		2
Have you suffered psychological stress or acute disease in the past 3 months?	Yes		0
	No		2
Neuropsychological problems	Severe dementia or depression		0
	Mild dementia		1
	No psychological problems		2
Body Mass Index (BMI)	< 19		0
	19 to less than 21		1
	21 to less than 23		2
	>= 23		3
Lives in own home (not care home)	No		0
	Yes		1
Takes more than 3 prescription drugs per day	Yes		0
	No		1
Pressure sores or skin ulcers	Yes		0
	No		1
How many full meals does the patient eat daily?	1 meal		0
	2 meals		1
	3 meals		2
Selected consumption markers for protein intake	• At least one serving of dairy products per day<br>• Two or more servings of legumes or eggs per day<br>• Meat, fish, or poultry every day	0 or 1 yes	0.0
		2 yes	0.5
		3 yes	1.0
Consumes two or more servings of fruit or vegetables per day?	No		0
	Yes		1
How much fluid (water, juice, coffee, tea milk ….) is consumed per day?	Less than 3 cups		0.0
	3 to 5 cups		0.5
	More than 5 cups		1.0
Mode of feeding	Unable to eat without assistance		0
	Self-fed with some difficulty		1
	Self-fed with no difficulty		2
Self-view of nutritional status	Views self as being malnourished		0
	Is uncertain of nutritional state		1
	Views self as having no nutritional problem		2
	Not as good		0.0
	Does not know		0.5

In comparison with other people of the same age, how does the patient consider his / her health status?	As good	1.0
	Better	2.0
Mid-arm circumference (MAC) in cm Specify: ________	Less than 21	0.0
	21 to 22	0.5
	Greater than 22	1.0
Calf circumference (CC) – widest part of the calf in cm Specify: ________	Less than 31	0
	31 or greater	1

Total score: /30

Screening (short form):

0-7: Malnourished   8-11: At risk of malnutrition   12-14: Normal nutritional status

Assessment (full form):

0-16.5: Malnourished   17-23.5: At risk of malnutrition   24-30: Normal nutritional status
