# Supplementary material for: Development of a UK core dataset for geriatric medicine research: a position statement and results from a Delphi consensus process
Source: BMC Geriatr. 2023 Mar 23;23:168. doi: 10.1186/s12877-023-03805-5 (PMC10035483; doi:10.1186/s12877-023-03805-5)
Supplement: Supplementary file 2 — Additional file 2. Questionnaires used in Step 2, 3, and 4 of the Delphi study. [file 12877_2023_3805_MOESM2_ESM.pdf]

# Core geriatric medicine dataset - Delphi process (Round 1)

You are being invited to take part in this survey as an active researcher in the field of geriatric medicine. You are being invited either following attendance at the BRC-Newcastle Academic Geriatric Medicine Workshop or as an expert within the field. The purpose behind this is to agree a set of core measurements and assessments that we agree should be included in geriatric medicine research and to ensure standardisation of approach, and potentially enable future sharing of datasets, tissue samples and collaboration as appropriate. We consider this core dataset to be of relevance to all geriatric medicine research from fundamental translational research through to clinical trials.

This Delphi process will continue until stability is reached, with a minimum of three rounds. Your participation in this process is entirely voluntary. We are collecting details of who has completed the survey so that we have knowledge of who has participated - everyone who participates in this process will be included as a collaborative author on publications arising from this process as a minimum. By completing this survey you agree to the transfer of your data (name and email address) to the University of Birmingham. All data will be held securely in line with the Data Protection Act 2018.

The first round of this process has been kept deliberately broad. Future rounds will include increasing levels of specificity until stability is reached. The final core dataset will be developed from this process with agreement for an expert committee and involvement of older adults to reach a consensus. In this current round, if you consider any elements of the proposed core dataset that should not be included at all then please specify this in the comments. We appreciate that some aspects may not be possible to include in all studies, but we consider that including all aspects will ensure that where these are measured, that our assessments are standardised.

For further information, please email: t.jackson@bham.ac.uk or c.welch@bham.ac.uk

* Required

1. Name *

______________________________

2. Email address *

______________________________

3. What are your thoughts on the inclusion of participants who lack capacity to consent in geriatric medicine research, either at time of recruitment, or if they later lose capacity during the research process?

4. What information do you think should be included as part of basic demographics?

5. How should multi-morbidity be recorded e.g. free text or scale? Do you recommend any specific scale?

6. How should functional dependency be recorded? Do you recommend any specific scale for basic and/ or instrumental activities of daily living?

7. How should nutrition or risk of malnutrition be assessed?

8. What measures of frailty should be included?

9. If Fried included, how should we quantify energy expenditure and/ or physical activity?

10. If standardised FI included, how many variables should be included?

11. Please suggest variables that should be included within a standardised FI

12. What measures of body morphology (e.g. height, weight, BMI, abdominal to hip circumference ratio) should be included?

13. How should grip strength be measured (e.g. Dominant hand? Average of multiple readings? Best of any reading in any hand? Is it necessary to use a Jamar?)

14. How should usual gait speed be measured e.g. distance of course? Is it necessary to build up speed first?

15. How should cognition be measured?

16. How should mood be measured?

17. How should delirium be assessed for?

18. Any specific requirements for storing of plasma and/ or serum or other samples?

19. How should muscle quantity and/or quality be measured?

20. Any other thoughts/ anything else that should be included not otherwise mentioned above?

______________________________________________
______________________________________________
______________________________________________
______________________________________________
______________________________________________

Thank you for taking the time to complete this survey.

We will be in touch in the future for future rounds of this Delphi process, which we consider will help to strengthen geriatric medicine research within the UK.

This content is neither created nor endorsed by Google.

Google Forms

# Delphi process (Round 2)

You are being invited to take part in this survey as an active researcher in the field of geriatric medicine. You are being invited either following attendance at the BRC-Newcastle Academic Geriatric Medicine Workshop, following completion of Round 1 of our Delphi process, or as an expert within the field. The purpose behind this is to agree a set of core measurements and assessments that we agree should be included in geriatric medicine research and to ensure standardisation of approach, and potentially enable future sharing of datasets, tissue samples and collaboration as appropriate. We consider this core dataset to be of relevance to all geriatric medicine research from fundamental translational research through to clinical trials. There are two aspects to this:

Reaching a consensus as to which assessment tools and information we agree should be considered "core" and included in all important geriatric medicine researchReaching a consensus as to how assessments should be performed and information recorded, regardless of whether or not this is "core" to enable standardisation across sites

This Delphi process will continue until stability is reached, with a minimum of three rounds. Your participation in this process is entirely voluntary. We are collecting details of who has completed the survey so that we have knowledge of who has participated - everyone who participates in this process will be included as a collaborative author on publications arising from this process as a minimum. By completing this survey you agree to the transfer of your data (name and email address) to the University of Birmingham. All data will be held securely in line with the Data Protection Act 2018. We are collecting survey responses through REDCap, which is a secure encrypted database, hosted by the University of Birmingham. The University of Birmingham's Data Protection policy can be found here: https://www.birmingham.ac.uk/university/governance/policies-regs/data-protection.asp

The final core dataset will be developed from this process with agreement for an expert committee and involvement of older adults to reach a consensus. The second round of this process has been developed using responses from those who completed Round 1 of the Delphi process. We ask that all responses to this current round could please be completed by 31st October 2019 - if you envisage any problems with this deadline then please contact us directly.

For further information, or if you wish to unsubscribe, please email: t.jackson@bham.ac.uk or c.welch@bham.ac.uk

---

1) Name

________________________________

---

2) Email address

________________________________

3) Which of these aspects (regardless of how they they are recorded) do you consider to be "core" and required for important geriatric medicine research? Please tick all items that you think should be considered "core".

- ☐ Basic demographics
- ☐ Multi-morbidity
- ☐ Functional dependency
- ☐ Nutrition
- ☐ Frailty
- ☐ Body morphology
- ☐ Handgrip strength
- ☐ Usual gait speed
- ☐ Other physical performance measures e.g. SPPB
- ☐ Cognition
- ☐ Mood
- ☐ Delirium
- ☐ Specific requirements for storing biological specimens
- ☐ Muscle quantity and/or quality
- ☐ Quality of life
- ☐ Medication count
- ☐ Behavioural and Psychotic Symptoms
- ☐ Pain
- ☐ Data from caregivers - validated informant versions
- ☐ Place of residence
- ☐ Walking aids used
- ☐ Visual and hearing impairment
- ☐ Falls over last year
- ☐ Continence
- ☐ Admissions over last year
- ☐ Loneliness

4) Which of these items should be included in basic demographics? Please tick all that apply.

- ☐ Age
- ☐ Gender
- ☐ Ethnicity
- ☐ Level of education
- ☐ Previous occupation
- ☐ Nationality
- ☐ Marital status
- ☐ Social deprivation index
- ☐ Postcode
- ☐ Number of cars available to household
- ☐ Age left formal education
- ☐ Socioeconomic status
- ☐ Biological sex (as opposed to or in addition to gender)
- ☐ Smoking status (current/ex/non and pack years)
- ☐ Alcohol use (units/weeks)

5) How should multimorbidity be recorded? Please choose your preferred option

- ◯ Free text only
- ◯ Free text and count/scale
- ◯ Count/scale only

6) If using a multimorbidity scale, which should be used?

- ◯ UK Biobank hierarchy of ~450 conditions
- ◯ Charlson comorbidity scale
- ◯ Geriatric Index of Comorbidity
- ◯ Specific diagnostic ICD-10 codes
- ◯ Multimorbidity count
- ◯ Binary 'yes/no' for long-term conditions and multimorbidity count
- ◯ Cumulative illness rating scale
- ◯ Embedded within frailty index

7) How should basic Activities of Daily Living be recorded?

◯ Barthel index
◯ Katz ADLs
◯ As per ELSA
◯ OPCS scale for disability

8) How should instrumental ADLs be recorded?

◯ Nottingham extended ADLs
◯ Lawton ADLs
◯ As per ELSA

9) How should risk of malnutrition be recorded?

◯ MUST
◯ MNA (short form)
◯ Weight and BMI alone
◯ SCREEN II
◯ SNAQ

10) If a more thorough assessment of nutritional status is being performed, how should this be recorded?

◯ MNA (full form)
◯ Subjective global assessment

11) If you consider measurement of frailty to be "core", which of the following measures do you think should be included (tick all that apply)?

☐ Fried frailty phenotype
☐ Frailty index
☐ Clinical frailty scale
☐ PRISMA 7 questionnaire
☐ Edmonton frail scale

12) Considering the Fried phenotype, where used, how should energy expenditure/ physical activity be quantified?

◯ As per frailty intervention trial (yes to any of the following over last 3 months - no weightbearing activity, more than 4 hours/day sitting, short walk less than once/month)
◯ Rapid assessment of physical activity
◯ As per SHARE protocol ("How often do you engage in activities that require a low or moderate level of energy such as gardening, cleaning the car, or taking a walk?" Responses were coded as follows: 1="More than once a week"; 2="Once a week"; 3=One to three times a month" and 4="Hardly ever or never")
◯ Physical activity scale for the elderly

13) Which of these would you preference to use as a standardised frailty index?

◯ As per electronic Frailty Index (adapted for secondary care use if applicable) - 36 item
◯ As per ELSA - 60 item
◯ As per original CSHA - 70 item
◯ We should use a Delphi approach to arise at a a new standardised FI for use across populations, which may include items taken from the indices mentioned above

14) What measures of body morphology should be included? (tick all that apply)

☐ Height
☐ Weight
☐ BMI
☐ Waist: hip circumference ratio

15) Considering measurment of handgrip strength, do you agree that all research should use only Jamar dynamometers to ensure standardisation?

◯ Yes
◯ No

16) What protocol should be used when measuring handgrip strength?

- ◯ Best of three - dominant hand only
- ◯ Two readings on both sides; best of all readings
- ◯ Best of two - dominant hand only
- ◯ Three readings on each side: best of all readings
- ◯ Measure dominant and non-dominant readings and take the average of multiple readings on each side

17) What distance of course should be used for measurement of usual gait speed?

- ◯ 3m
- ◯ 4m
- ◯ 5m
- ◯ 10m

18) When measuring usual gait speed, should "start" and "stop" be performed when participant is actively walking (i.e. walking 1m before and after start of course)

- ◯ Yes
- ◯ No

19) How should physical performance be measured (regardless of whether or not this is considered core)?

- ◯ Usual gait speed only
- ◯ Short physical performance battery to include usual gait speed
- ◯ 6 minute walk distance
- ◯ Timed up and go
- ◯ Chair stands only

20) How should cognition be measured?

- ◯ MoCA
- ◯ AMTS - AMT10
- ◯ MMSE
- ◯ ACE-III
- ◯ Stroop test
- ◯ AMT4 (used as part of 4AT)
- ◯ IQCODE-SF (proxy screen)
- ◯ Colour trails
- ◯ Fluency and clock drawing taken from ACE-III (potential to expand to complete ACE-III if relevant to research)

21) How should mood be measured?

- ◯ HADS
- ◯ GDS-1
- ◯ GDS-4
- ◯ GDS-5
- ◯ GDS-15
- ◯ Simple questioning: is there a history of a mood disorder?

22) How should delirium be assessed for?

- ◯ 4AT
- ◯ 4AT then DSM-5
- ◯ SqID
- ◯ SqID then DSM-5
- ◯ DSM-5 alone (no need for screening tool)
- ◯ CAM

23) Which of these requirements for storing of biological samples should be standardised (tick all that apply)?

- ☐ Store in a link anonymised manner linked to core dataset
- ☐ Freeze at -20 (or lower)
- ☐ Freeze at -70
- ☐ Freeze at -80
- ☐ Ethics to allow samples to be moved to other centres
- ☐ Storage of peripheral blood mononuclear cells in addition to serum and plasma
- ☐ Storage of some specimens in plates and some in cryovials

24) How should muscle quantity and/or quality be measured to allow standardisation across sites (please select the simplest acceptable measure)?

- ◯ US - bilateral anterior thigh thickness as per Wilson et al
- ◯ US - rectus femoris cross-sectional area
- ◯ BIA - impedance, reactance recorded alongside height, weight, and gender, as well as any specific results dependent on the device and protocol
- ◯ DXA
- ◯ MRI
- ◯ CT

25) How should quality of life be recorded (new to round 2)?

__________________________________

26) How should pain be recorded (new to round 2)?

__________________________________

27) How should continence be recorded (new to Round 2)?

__________________________________

28) How should loneliness be recorded?

__________________________________

29) How should behavioural and psychotic symptoms be recorded?

__________________________________

# Delphi Round 3

You are being invited to take part in this survey as an active researcher in the field of geriatric medicine. The purpose behind this is to agree a set of core measurements and assessments that we agree should be included in geriatric medicine research and to ensure standardisation of approach, and potentially enable future sharing of datasets, tissue samples and collaboration as appropriate. We consider this core dataset to be of relevance to all geriatric medicine research from fundamental translational research through to clinical trials. There are two aspects to this:

Reaching a consensus as to which assessment tools and information we agree should be considered "core" and included in all important geriatric medicine researchReaching a consensus as to how assessments should be performed and information recorded, regardless of whether or not this is "core" to enable standardisation across sites

This Delphi process will continue until stability is reached, with a minimum of three rounds. Your participation in this process is entirely voluntary. This is the third round in the Delphi process and we have deliberately chosen to keep this round anonymous. We are collecting survey responses through REDCap, which is a secure encrypted database, hosted by the University of Birmingham. The University of Birmingham's Data Protection policy can be found here: https://www.birmingham.ac.uk/university/governance/policies-regs/data-protection.asp

The final core dataset will be developed from this process with agreement for an expert committee and involvement of older adults to reach a consensus. The third round of this process has been developed using responses from those who completed Round 1 and 2 of the Delphi process. We ask that all responses to this current round could please be completed by March 31st 2020 - if you envisage any problems with this deadline then please contact us directly.

For further information, or if you wish to unsubscribe, please email: t.jackson@bham.ac.uk or c.welch@bham.ac.uk

## Items included

1) The following items were considered to constitute core items in geriatric medicine research by >80% of participants in Round 2. Do you agree to inclusion of each of these items as core to all geriatric medicine research? Please select all that you agree to:

- ☐ Basic demographics - age, gender, ethnicity
- ☐ Multi-morbidity
- ☐ Functional dependency
- ☐ Frailty
- ☐ Cognition
- ☐ Medication count
- ☐ Place of residence

2) If you did not agree to any of the above, then please describe your rationale below:

______________________________________

3) Please select all items that should be included as optional items, with specific recommendations as to how these are recorded where appropiate:

- ☐ Highest level of education
- ☐ Smoking status
- ☐ Alcohol intake
- ☐ Marital status
- ☐ Social deprivation index
- ☐ Previous occupation
- ☐ Socioeconomic status
- ☐ Nationality
- ☐ Postcode
- ☐ Nutrition
- ☐ Body morphology (height, weight, BMI)
- ☐ Handgrip strength
- ☐ Usual gait speed
- ☐ Mood
- ☐ Delirium
- ☐ Quality of life
- ☐ Continence
- ☐ Walking aids used
- ☐ Visual and hearing impairment
- ☐ Falls over last year
- ☐ Admissions over last year

4) Please add any comments related to the above (items selected/ non-selected) below:

__________________________________

**Multimorbidity**

**Please rank the following options  for recording of multimorbidity from 1 (least preferable) to 5 (most preferable):**

| | 1 | 2 | 3 | 4 | 5 |
|---|---|---|---|---|---|
| 5) Free text only | ○ | ○ | ○ | ○ | ○ |
| 6) Charlson Comorbidity Scale | ○ | ○ | ○ | ○ | ○ |
| 7) Comorbidity count | ○ | ○ | ○ | ○ | ○ |
| 8) Free text and Charlson Comorbidity Scale | ○ | ○ | ○ | ○ | ○ |
| 9) Free text and Comorbidity Count | ○ | ○ | ○ | ○ | ○ |

**Functional dependency**

**Do you agree to recording of functional dependency in the following ways:**

| | Yes | No |
|---|---|---|
| 10) Basic Activities of Daily Living by Barthel Index | ○ | ○ |
| 11) Instrumental Activities of Daily Living by Nottingham Extended Activities of Daily Living | ○ | ○ |

## Frailty

**Please rank the following options for recording of frailty from 1 (least preferable) to 7 (most preferable). Please bear in mind that handgrip strength and gait speed have not otherwise been considered "core" at present. How these are measured will be re-discussed in Round 4 (consensus discussion at meeting):**

| | | 1 | 2 | 3 | 4 | 5 | 6 | 7 |
|---|---|---|---|---|---|---|---|---|
| 12) | Fried frailty phenotype only | ◯ | ◯ | ◯ | ◯ | ◯ | ◯ | ◯ |
| 13) | Clinical frailty scale (CFS) only | ◯ | ◯ | ◯ | ◯ | ◯ | ◯ | ◯ |
| 14) | Frailty index (FI) only | ◯ | ◯ | ◯ | ◯ | ◯ | ◯ | ◯ |
| 15) | Fried phenotype and CFS | ◯ | ◯ | ◯ | ◯ | ◯ | ◯ | ◯ |
| 16) | Fried phenotype and FI | ◯ | ◯ | ◯ | ◯ | ◯ | ◯ | ◯ |
| 17) | CFS and FI | ◯ | ◯ | ◯ | ◯ | ◯ | ◯ | ◯ |
| 18) | Fried, CFS, and FI | ◯ | ◯ | ◯ | ◯ | ◯ | ◯ | ◯ |

## Cognition

| | | Yes | No |
|---|---|---|---|
| 19) | Do you agree to recording of cognition by MoCA? | ◯ | ◯ |

## Optional aspects

20) How should nutritional screening be recorded?

◯ Malnutrition Universal Screening Tool (MUST) ◯ Mini-Nutritional Assessment Short Form (MNA-SF)

21) In Round 2, all who considered how nutritional assessment should be performed preferenced Mini-Nutritional Assessment (Full Form). Do you agree with this?

◯ Yes ◯ No

22) Multiple similar but differing protocols for handgrip strength were ventured. Most considered that the best of all readings, rather than an average reading should be used. In your opinion, should handgrip strength be measured:

◯ Dominant side only
◯ Both sides

23) How many times should handgrip strength be measured (as a minimum):

◯ 2 each side (4 total if both sides)
◯ 3 each side (6 total if both sides)

## Usual gait speed

**Measurement of usual gait speed was split between different options in Round 2. We are looking to standardise this measurement in a way that can be easily used in a variety of settings (home visits, clinic, ward setting). Usual gait speed may be used as part of Fried frailty phenotype, if this is included. Please rank the options below from 1 (least preferable) to 8 (most preferable). "Active walking" refers to active "start" and "stop" whilst the participant is walking:**

| | 1 | 2 | 3 | 4 | 5 | 6 | 7 | 8 |
|---|---|---|---|---|---|---|---|---|
| 24) | | | | | | | | |

| | | | | | | | | |
|---|---|---|---|---|---|---|---|---|
| 3metres from stationary | ◯ | ◯ | ◯ | ◯ | ◯ | ◯ | ◯ | ◯ |
| 25) 3metres with 1metre active walking | ◯ | ◯ | ◯ | ◯ | ◯ | ◯ | ◯ | ◯ |
| 26) 4 metres from stationary | ◯ | ◯ | ◯ | ◯ | ◯ | ◯ | ◯ | ◯ |
| 27) 4 metres with 1 metre active walking | ◯ | ◯ | ◯ | ◯ | ◯ | ◯ | ◯ | ◯ |
| 28) 5 metres from stationary | ◯ | ◯ | ◯ | ◯ | ◯ | ◯ | ◯ | ◯ |
| 29) 5 metres with 1 metre active walking | ◯ | ◯ | ◯ | ◯ | ◯ | ◯ | ◯ | ◯ |
| 30) 10 metres from stationary | ◯ | ◯ | ◯ | ◯ | ◯ | ◯ | ◯ | ◯ |
| 31) 10 metres with 1 metre active walking | ◯ | ◯ | ◯ | ◯ | ◯ | ◯ | ◯ | ◯ |

32) Do you agree to recording of mood by the Geriatric Depression Scale 15 (GDS-15)? ◯ Yes ◯ No

33) Do you agree to recording of delirium status by 4AT? ◯ Yes ◯ No

34) How should quality of life be measured? ◯ EQ5D ◯ SF36

35) Do you agree that recording of continence as part of Barthel index is sufficient? ◯ Yes ◯ No

36) Do you have any additional comments you wish to make?

__________________________________________
